# Accessibility to Primary Care Services for Immigrants Experiencing Homelessness in England: A Qualitative Exploratory Study

**DOI:** 10.3390/ijerph23060726

**Published:** 2026-05-29

**Authors:** Carol Namata

**Affiliations:** 1 Faculty of Medicine, Health, and Social Care, Canterbury Christ Church University, Canterbury CT1 1QU, UK; cn397@canterbury.ac.uk; 2 Faculty of Health Studies, Norquest College, Edmonton, AB T5J 1L6, Canada

**Keywords:** access to primary care, homeless immigrants, United Kingdom

## Abstract

**Highlights:**

**Public health relevance—How does this work relate to a public health issue?**

- Immigrants experiencing homelessness face intersecting structural barriers that impede access to primary care services and early mental health intervention.
- Digitalisation of primary care risks further excluding populations with low digital literacy or connectivity.

**Public health significance—Why is this work of significance to public health?**

- Access to primary care is shaped by overlapping social vulnerabilities that reinforce exclusion among marginalised populations.
- Restricted access shifts care-seeking toward emergency services, contributing to poorer health outcomes and increased health system burden.

**Public health implications—What are the key implications or messages for practitioners, policymakers and/or researchers in public health?**

- Improving access to primary care is critical for enabling early mental health interventions and reducing reliance on crisis-driven emergency care.
- Primary care systems should adopt culturally responsive, trust-based, and inclusive approaches to ensure equitable access to healthcare.

**Abstract:**

Access to primary care services is essential for promoting mental health, yet immigrants experiencing homelessness face significant barriers to care. This study explores factors that influence access to primary care services in the UK. A qualitative design was employed, involving in-depth semi-structured interviews with 30 immigrants experiencing homelessness and 30 stakeholders across healthcare, voluntary, and local authority sectors. Data were analysed using thematic analysis, guided by the Levesque framework of healthcare access and an intersectionality lens. Findings reveal that access is influenced by intersecting structural barriers, including fear of detention and deportation, cultural stigma surrounding mental health, digital exclusion, and financial hardship. These barriers delay help-seeking and shift care-seeking toward emergency services. Increasing reliance on digital systems in primary care further excludes individuals with limited access to devices, connectivity, or digital skills. These findings indicate that barriers to accessing primary care services may hinder the early identification and preventive management of mental health needs among homeless immigrants. Improving access requires structural reforms that address legal, financial, and digital barriers, alongside more culturally responsive and trust-based care. Without such changes, digital health innovations risk reinforcing existing inequalities and limiting the role of primary care in early mental health intervention and prevention.

## 1. Introduction

Access to healthcare is a fundamental human right [1], yet for many marginalised populations, including asylum seekers and refugees, persistent structural barriers embedded within healthcare and immigration systems [2] continue to undermine equitable access to healthcare. Immigrants often have complex physical and mental health needs, shaped by experiences in their country of origin, their migration journey, their host country’s entry and integration policies, and living and working conditions. These experiences can increase the vulnerability of refugees and migrants to chronic and infectious diseases [3], thereby emphasising the critical importance of timely, appropriate, and equitable access to healthcare services. These health needs rarely diminish upon arrival in their host country; rather, they are often intensified by the complexity of navigating an unfamiliar health system, pervasive cultural and linguistic barriers, and persistent fears surrounding immigration enforcement [4]. As such, the health inequalities experienced by immigrant communities are not merely inherited from pre-migration contexts but are actively reproduced through the institutional, social, and policy environments of the host country [5].

In the United Kingdom (UK), primary care services (PCSs) serve as the main entry point to the National Health Service (NHS), positioning general practitioners and other community-based professionals as key gatekeepers to care [6]. The NHS is the publicly funded healthcare system in England and provides a wide range of health services, including general practitioner (GP) care, hospitals, pharmacies, mental health services, prescriptions, vaccination, and emergency care. It aims to give people access to healthcare based on need rather than ability to pay [7]. Beyond this role, PCSs are central to public mental health promotion, enabling early identification, timely intervention, and coordinated management of mental health conditions. As the first point of contact for many individuals experiencing psychological distress, primary care supports holistic, person-centred care, improves diagnostic efficiency, and reduces delays in treatment. It also facilitates integrated care planning and continuity across services, contributing to improved outcomes and reduced reliance on more resource-intensive emergency and secondary care [8].

Furthermore, PCSs regulate entry to most specialist services, which, apart from limited exceptions, such as genitourinary medicine, generally require general practitioner (GP) referral. As a result, the structure and functioning of primary care critically shape individuals’ ability to navigate the NHS and obtain timely specialist care [9]. However, existing research demonstrates that PCSs often operate as sites in which broader structural inequities become acutely visible. About 16% of the UK population (10.7 million people in 2022) was born abroad, and more recent data indicate that about 171,000 individuals migrated to the UK for the long term in 2025, defined as moving for 12 months or more [10,11]. Immigrants routinely face barriers to accessing PCSs, such as limited access to interpretation services, language barriers, financial hardship related to prescription charges, dental fees, and transport costs, as well as discriminatory treatment linked to race, religion, or immigration status [12,13,14]. Consequently, many immigrants tend to rely on emergency departments for conditions better managed within primary care, a pattern associated with poorer health outcomes and increased strain on emergency services [15]. For people experiencing homelessness, barriers to accessing healthcare are further amplified by structural vulnerabilities. Similarly to immigrant populations, homeless individuals encounter multiple barriers to accessing PCSs, including stigma, discrimination, limited trust in providers, and inadequate communication or information sharing. These barriers often delay care-seeking until conditions become advanced and complex. This challenge is further intensified in the digital era, where access to primary care is increasingly mediated through telephone and online appointment systems, effectively shifting initial contact into digital spaces [16]. For individuals experiencing homelessness, limited access to devices, connectivity, and digital literacy [17] may act as an additional structural barrier, further hindering access to PCSs. Consequently, people experiencing homelessness also demonstrate higher rates of emergency department use and acute hospital admissions, as well as longer lengths of stay due to co-occurring physical, mental health, and social needs. These factors lead to substantially poorer health and social care outcomes compared with the general population [18].

By December 2025, an estimated 3536 people in England were sleeping rough (i.e., homeless) on a single night [19]. The intersection of homelessness and immigration status generates a compounded form of marginalisation. Despite the significance of this intersection, research specifically examining PCS access among immigrants experiencing homelessness in England remains sparse. Much of the existing literature views migration [12,13,14] and homelessness [20,21,22] as discrete categories, thereby overlooking how these intersecting social identities produce barriers to accessing PCSs. This approach has resulted in an inadequate understanding of the specific mechanisms through which immigrants experiencing homelessness navigate, experience, and are excluded from healthcare systems. Crucially, from a public mental health perspective, primary care serves as a vital platform for early intervention [23]. For vulnerable populations, timely engagement with general practitioners can act as a protective factor, supporting psychological wellbeing and preventing the escalation of distress into acute crises [23]. When access to primary care is constrained, however, care-seeking becomes increasingly crisis-driven, with individuals presenting to emergency services at advanced stages of need [24]. This reactive model is clinically suboptimal and contributes to health system pressures.

This study addresses this gap by examining access to primary care services, drawing on perspectives from both immigrants experiencing homelessness and relevant stakeholders. In doing so, it also considers the implications of access barriers for mental health promotion, particularly how limited access to primary care shifts care-seeking toward crisis-driven emergency services. Guided by Levesque et al.’s access framework [25] together with an intersectionality lens [26], this study explores how the intersection of social identities such as immigration status and homelessness creates inequalities that hinder access to PCSs. This combined approach reveals how systemic inequities are reproduced at the point of care and how the accessibility of PCSs is conditioned by the social and legal positions of homeless immigrants in England.

## 2. Materials and Methods

### 2.1. Research Design

This study employed a qualitative research design to explore the provision of and access to PCSs among immigrants experiencing homelessness in England. A qualitative approach was chosen to capture rich, in-depth accounts of participants’ lived experiences and perspectives, allowing for a nuanced understanding of the social, structural, and institutional factors shaping access to primary care [27]. The reporting of this qualitative study follows the Consolidated Criteria for Reporting Qualitative Research (COREQ) checklist [28] (Supplementary Materials).

### 2.2. Study Participants

Primary qualitative data were collected over a five-month period. The study population comprised two participant groups: (1) immigrants experiencing homelessness (*N* = 30) and (2) stakeholders involved in the provision of or facilitation of access to PCSs for this population (*N* = 30). Homeless immigrant participants were adults aged 18 years and above and included individuals with diverse immigration statuses, such as asylum seekers and refugees, as illustrated in Table 1. Stakeholders were recruited from the voluntary sector, healthcare services, and local authorities, and included both frontline practitioners and managerial staff, as presented in Table 2.

### 2.3. Sampling Procedures and Sample Size

A multi-method sampling strategy involving purposeful and snowball sampling was used to recruit homeless immigrants and stakeholders in the UK. Homeless immigrant participants were recruited through voluntary sector organisations such as community shelters and non-profit organisations, while stakeholders were identified through professional networks within healthcare services, local authorities, and third-sector organisations. Snowball sampling enabled the inclusion of participants who were difficult to reach due to homelessness or distrust of formal institutions.

### 2.4. Data Collection

Data were collected through in-depth, semi-structured interviews conducted via Microsoft Teams 1.5 (Microsoft Corporation, Redmond, WA, USA), (video or audio) or via phone calls. Interviews were audio-recorded and lasted approximately 45–60 min each. Separate interview guides were used for homeless immigrant participants and stakeholders, and were designed to explore perspectives and experiences navigating primary care services. All interviews were conducted in English. Participants were provided with a modest honorarium in appreciation for their time and contribution to the study.

### 2.5. Levesque Framework and Intersectionality Lens

This study utilised the Levesque Framework of Patient-Centred Access to Healthcare in conjunction with an intersectionality lens to examine the barriers to PCSs for immigrants experiencing homelessness. The analysis specifically focused on the five supply-side dimensions of the Levesque framework: approachability, acceptability, availability and accommodation, affordability, and appropriateness [25]. Approachability relates to the extent to which individuals can identify that healthcare services exist, can be reached, and may positively impact their health and wellbeing. Acceptability concerns the cultural and social factors that influence whether healthcare services are perceived as appropriate and respectful of patients’ values, beliefs, gender, language, and cultural background. Availability and accommodation relate to the physical existence of healthcare resources and the organisation of services, including opening hours, appointment systems, location, and the ability of services to accommodate patients’ needs. Affordability refers to economic capacity of individuals to spend resources and time to access healthcare services, including direct costs, transportation, and opportunity costs. Appropriateness refers to the extent to which healthcare services adequately meet patients’ needs through provision of timely, high-quality and coordinated care. These dimensions characterise the health system’s capacity to provide care and its responsiveness to the needs of marginalised populations. While the Levesque framework provides a structured approach to understanding how healthcare systems enable or constrain access, it does not fully account for how overlapping social positions shape individuals’ experiences within these systems. To address this limitation, an intersectionality lens was applied to examine how multiple, co-existing social identities such as immigration status, homelessness, gender, race/ethnicity, and poverty interact within broader systems of power to produce compounded disadvantage [29,30]. From this perspective, immigrants experiencing homelessness are not treated as a homogeneous group; rather, their access to care is understood as relational and shaped by intersecting structural inequities. Integrating these frameworks enables a more nuanced and equity-focused analysis of access. Specifically, it allows for examination not only of how healthcare systems are structured (as captured by Levesque’s dimensions), but also of how these structures are differentially experienced depending on individuals’ social and legal positioning. This combined approach provides deeper insight into the mechanisms through which access barriers are produced and sustained, and highlights the need for contextually tailored strategies to reduce disparities among immigrants experiencing homelessness.

### 2.6. Data Analysis

All interviews were audio-recorded with participants’ consent. Interviews conducted via Microsoft Teams 1.5 (Microsoft Corporation, Redmond, WA, USA), (audio/video) were transcribed using Microsoft Teams’ built-in transcription function, while telephone interviews were transcribed using Otter.ai 2022 (Otter.ai, Inc., Mountain View, CA, USA). All transcripts were then carefully checked against the original recordings to ensure accuracy and completeness prior to analysis. Data were analysed using reflexive thematic analysis, following Braun and Clarke’s six-phase approach [31]. Analysis was iterative and inductive, identifying patterns and themes relating to immigrants’ experiences of accessing primary care and stakeholders’ perspectives on service provision. The analytic process began with familiarisation, where a researcher (C.N.) thoroughly read the transcripts to gain a deep understanding of the content. This led to the iterative process of generating initial codes for common words, phrases, and concepts. Codes were managed, organised, and tracked using NVivo 13 software (Lumivero/QSR International, Burlington, MA, USA). The codes were reviewed for patterns and relationships, then grouped into coherent subthemes, which were subsequently synthesised into broader, overarching themes. Themes were interpreted using Levesque et al.’s healthcare access framework, organising findings across approachability, acceptability, availability and accommodation, affordability, and appropriateness [25]. An intersectionality lens further informed interpretation by examining how overlapping social identities and structural positions, including immigration status, homelessness, gender, race/ethnicity, and poverty, interacted to shape experiences of access to primary care services. To enhance trustworthiness, a senior researcher independently reviewed a subset of transcripts and the coding framework, providing feedback that informed further theme refinement. Data saturation was assessed and confirmed when no new themes emerged. Anonymised participant quotations are presented in the Results section to illustrate and substantiate the identified themes.

### 2.7. Ethical Considerations

Ethical approval for this study was obtained from the Research Ethics Committee of Canterbury Christ Church University (protocol code: ETH2223-0076 and date of approval: 22 October 2021). Prior to participation, individuals received comprehensive written and verbal information regarding the study’s objectives, procedures, potential benefits, risks, confidentiality measures, expected duration of the interview, the voluntary nature of participation, and their right to withdraw at any time without consequence. Written informed consent was obtained from each participant before the interview, and verbal informed consent was obtained when requested by individual participants. To ensure confidentiality and anonymity, all responses were assigned numbers, and all potentially identifying information was meticulously removed from transcripts. The collected data were stored securely on a password-protected university drive, accessible only to the authorised research team members.

## 3. Results

Table 1 summarises the characteristics of immigrant participants experiencing homelessness (*N* = 30). Participants were predominantly female (66.7%) and represented a wide age range, with most aged between 30 and 49 years. The majority identified as African, followed by Arab, Asian and Caribbean ethnicities, and had lived in the UK for several years, with over half reporting a length of stay of ten years or more. Most participants were asylum seekers or refugees at the time of the interview, reflecting varying and often insecure immigration statuses. Table 2 presents the characteristics of stakeholders (*N* = 30), who were primarily drawn from the voluntary sector, healthcare services and local councils, with females comprising nearly two-thirds of the sample. Stakeholders occupied diverse frontline and managerial roles, including project workers, clinicians, social prescribers and local authority professionals, providing a broad range of perspectives on the provision of and access to PCSs for immigrants experiencing homelessness in England.

The findings are organised according to the five dimensions of the Levesque framework [25], which include approachability, acceptability, availability and accommodation, affordability, and appropriateness. Central to this analysis is an intersectionality lens which reveals that barriers to PCSs are not merely the result of a single factor such as homelessness or migrant status. Rather, access is shaped by the interaction of multiple, overlapping social positions which collectively hinder homeless immigrants’ access to PCSs.

### 3.1. Approachability

#### 3.1.1. Unfamiliarity with the Roles of the General Practitioners

Limited understanding of the role of GPs emerged as a key barrier among recently arrived immigrants experiencing homelessness. This lack of familiarity reflects the intersection of migration status, housing instability, and limited prior exposure to the UK healthcare system. Participants were often uncertain about the types of health concerns that could be addressed within primary care, which constrained their ability to navigate services effectively. This uncertainty was particularly evident in relation to mental health. Many participants were unsure whether GPs could provide support for psychological distress or facilitate access to counselling and specialist services. As a result, some sought support from charitable or community-based organisations, which were perceived as more accessible and better aligned with their immediate needs. This gap in understanding limits opportunities for early intervention and contributes to fragmented pathways of care. This uncertainty is illustrated by one asylum seeker:

*“I’ve never mentioned it to my GP because I don’t know what the GPs are capable of. Like, can you call your GP to recommend you to a counsellor? I don’t fully know the role of the GPs here, I’m not aware”*.

(Male asylum seeker 8)

The unfamiliarity with the roles of general practitioners among homeless immigrants was supported by insights from other healthcare providers. These healthcare providers reported that some immigrants misunderstood the role of NHS gatekeepers and bypassed GPs to directly access emergency services before being directed back to GPs. While this may be perceived as a faster route to care, it can result in longer waiting times and may not be the most appropriate level of care for their condition. This highlights the complexity of navigating the UK healthcare system, particularly for immigrants from countries with different healthcare systems, hence contributing to unmet expectations among homeless immigrants. As mentioned by one general practitioner:

*“I think a lot of them, their first contact is with emergency services and then they get sent back to us. But again, it’s about knowing what the services are, why can’t it be done from Accident and Emergency (A&E), and why does it have to be through a GP? And to be honest, I think, a lot of people … don’t understand what different services are for, and there are so many different services. So, it is quite a difficult thing to navigate it all”*.

(Female general practitioner)

#### 3.1.2. Fear of Being Detained and Deported

Fear of detention and deportation emerged as a significant barrier to accessing PCSs among immigrants experiencing homelessness, particularly those with irregular or uncertain immigration status. This fear reflects the intersection of homelessness and legal precarity, whereby unstable housing and insecure status heighten both perceived visibility and vulnerability to state surveillance. As a result, many participants avoided engaging with GP services, even when experiencing serious or chronic health conditions, leading to delayed care and the exacerbation of health problems. GP registration, in particular, was perceived as a formalised process that could expose immigration status. Concerns were intensified by requirements to disclose personal information, such as country of origin and date of arrival in the UK. Within a context of immigration enforcement, these administrative practices were experienced as intrusive and potentially threatening, reinforcing distrust in primary care services. More broadly, these findings illustrate how healthcare systems can be perceived as extensions of immigration control, transforming sites of care into spaces of risk. A social prescriber explained how such requirements contributed to fear among homeless immigrants:

*“I think there’s fear of the health services in general … There’s an element of it that if they register with a doctor’s surgery, that officially means that they might be put on the system and they might be asked why they’re here. I know on registration forms, the GP surgeries ask them for arrival dates in the UK, when they first got here, and where did they come from? where were they born? And that might be a bit scary for some people”*.

(Male social prescriber)

Furthermore, some homeless immigrants believed that personal information shared with GP practices would be passed on to the Home Office, leading to detention or deportation. This perception reflects how immigration status, homelessness, and mistrust of institutions intersected to shape views of primary care as a site of risk rather than support. For these individuals, accessing GP services was perceived as a potential pathway to arrest rather than healthcare, reinforcing avoidance of primary care services. As one refugee explained:

*“All those places are scary, to be honest, I just feel like they will pick my information and give it to the Home Office. Maybe they are working together, then they will pass you over, and then their staff will start calling and looking for me… That is why I never actually went to the GP”*.

(Female refugee 28)

### 3.2. Acceptability

#### 3.2.1. Cultural Beliefs

Cultural beliefs surrounding illness, particularly mental health, emerged as a significant barrier to accessing PCSs among immigrants experiencing homelessness. However, these beliefs did not operate in isolation; rather, they intersected with gender norms, migration status, and experiences of homelessness to shape help-seeking behaviours and access to PCSs. Mental illness was frequently perceived as a sign of weakness, “craziness,” or institutionalisation, contributing to denial and reluctance to seek professional support. These perceptions were reinforced by gendered expectations of masculinity, particularly among immigrant men, where cultural norms emphasising strength, self-reliance, and emotional restraint constrained the expression of psychological distress. Within the context of migration and homelessness, both of which are associated with heightened stigma and marginalisation, these norms further limited engagement with formal mental health support. These intersecting social positions produced layered barriers to care, where stigma related to mental illness was intensified by gender, cultural identity, and structural vulnerability, ultimately shaping patterns of delayed or foregone help-seeking. As one project worker explained:

*“I work with [mentions ethnicity], especially the men they are very resistant to tell you they have mental problems. They have depression but men cannot have mental problems, it’s a cultural thing. They have pride, ‘I am a man, I have to be strong even if I live on the street”*.

(Project worker)

Other stakeholders described how cultural difference intersected with identity to shape experiences of stigma within primary care settings. For instance, some immigrant women who wore hijabs felt feared being stereotyped due to cultural differences, which discouraged them from booking GP appointments. These accounts illustrate how religious and cultural identity, gender, and minority status interacted to create barriers to accessing PCSs. As one youth ambassador noted:

*“Some of our females wear hijab and this is something so common in our countries, but here it is not common. So, they feel shy, you know, to book an appointment with the GP because they feel that they would be stereotyped by others. You know this kind of feeling being ecstatic and shy at the same time, you just don’t want to do something because you feel you are different from the others”*.

(Youth ambassador 1)

Additionally, stakeholders reported that certain cultural norms regarding family authority further constrained access to primary care, particularly for women and children. In some cases, expectations that men act as sole decision-makers meant that women and children could not access healthcare without a husband’s or father’s permission. As mentioned by one stakeholder:

*“You cannot just go except when the husband says so. The child cannot just go to the health service without the father’s permission. So in that way, it has to be with the permission of the father … So that’s in a way, maybe affecting access. I remember we had to do COVID tests for some family, and the father was not around, they had to wait until their father came before we conducted tests”*.

(Public health specialist)

#### 3.2.2. Inadequate Culturally Responsive Healthcare Services

Inadequate culturally responsive PCS emerged as a significant barrier to access for homeless immigrants, shaped by the intersection of migration experiences, homelessness, trauma exposure, and linguistic differences. Stakeholders reported that some immigrants disengaged from PCSs that did not adequately reflect their cultural backgrounds or lived experiences, resulting in delayed diagnosis and treatment, particularly for mental health conditions. Concerns were raised about the appropriateness of generic counselling services for addressing the complex mental health needs of homeless immigrants. Stakeholders questioned whether counsellors primarily trained to support the local population could adequately respond to trauma related to forced migration, displacement, and violence. This highlights how service design that does not account for intersecting experiences of trauma, migration, and homelessness can limit the effectiveness of PCSs. As one GP explained:

*“I think if they wanted to, they can access this counselling. But whether that counselling would then be appropriate to their needs, I think, is the problem because they would just be referred to generic counsellors who are used to dealing with the problems of the local population, which, is mainly depression, anxiety, and that sort of thing … I don’t think the counsellors dealing with anxiety and depression would be able to manage the specific problems of trauma within immigrant communities”*.

(General practitioner)

Participants also described how limited cultural awareness among some healthcare professionals hindered access to primary care, particularly mental health services. Decisions were perceived to be made without sufficient understanding of immigrants’ lived experiences, including exposure to violence, detention, and family loss. Although some providers demonstrated surface-level cultural awareness, participants felt that deeper contextual understanding of the immigrant experience was often lacking. As one asylum seeker stated:

*“A lot of people making decisions who don’t even know what we need … people are locked up in poor conditions, some people beat up in police stations, …some people have seen their whole family murdered. So they don’t know how to deal with it”*.

(Male asylum seeker 20)

Furthermore, some homeless immigrants faced challenges in communicating with healthcare providers due to language barriers. They reported challenges with expressing themselves and being understood by doctors during GP appointments. The limited time slots for appointments were inadequate for homeless immigrants with language difficulties, leading to a perception that GP practices did not prioritise their needs. This affected their interaction with doctors, as some immigrants felt that doctors made assumptions about their illnesses and simply collected their prescriptions without meaningful engagement. One female refugee reported that:

*“They don’t care… time they slot, sometimes I can’t even express myself very well…When I first came in, it was difficult for me to express myself and they just assumed what they wanted to assume. Interacting is not interacting as you just go in there and get your medication and go”*.

(Female refugee 24)

### 3.3. Availability and Accommodation

#### 3.3.1. Digital Exclusion

Digital exclusion emerged as a significant barrier to accessing PCS among homeless immigrants, shaped by the intersection of homelessness, poverty, and immigration status. Participants reported limited or no access to digital devices, phone credit, and mobile data, which constrained their ability to contact GP practices and book appointments. For some immigrants, the absence of a mobile phone or insufficient credit prevented communication with GP surgeries altogether, restricting access to care. Participants also described how the increasing reliance on digital appointment systems within primary care disproportionately disadvantaged homeless immigrants. While online booking platforms were perceived as convenient for some patients, they were often inaccessible to those without smartphones, internet access, or sufficient data. These systems therefore reinforced exclusion for individuals already experiencing housing instability and financial hardship. As one asylum seeker explained:

*“Last time I used to call, and now they have got a system called patient access. So, I have to book in at 7:00 in the evening for when I want an appointment. Then again, it is good and bad at the same time because one, you need to have a mobile phone, two, you need to have data. So, it’s kind of a yes and no at the same time as well …”*.

(Male asylum seeker 20)

Financial constraints further intensified digital exclusion. Asylum seekers described having to prioritise essential needs such as food over phone credit and data due to extremely limited allowances. For those with caregiving responsibilities, particularly parents, these competing demands made digital connectivity unattainable, despite its importance for accessing primary care. This intersection of poverty and homelessness directly affected healthcare access. One asylum seeker articulated this dilemma:

*“There is no way I can remove money from the £35 which I am collecting for buying credit when I know that I have to feed my son, I have to eat. The network is not even a priority, food is the priority. Because as long as there is food, there is health”*.

(Female asylum seeker 26)

#### 3.3.2. Long Waiting Times for Appointments

The majority of the homeless immigrants expressed dissatisfaction with the long waiting times for GP appointments, reporting delays of one to two weeks or even cancellations. They felt compelled to wait despite their dissatisfaction, emphasising their lack of choice. Those residing in Home Office accommodation, particularly asylum seekers in reception or camp centres, faced even longer waiting times, as they required referrals from centre-attached health practitioners, making it more challenging for them to access GP services. As reported by one project officer:

*“For people who are asylum seekers in reception centres, it’s a bit difficult to be seen by a GP because they need to go through a health practitioner who is in the centre who would then refer them to a GP”*.

(Refugee project officer)

Other homeless immigrants also expressed dissatisfaction with the long waiting times for specialised care. It was reported that some immigrants had to wait for many months to be seen by a specialist. Some reported being tossed around from one service to the next without being attended to by a specialist. This created delays in obtaining appointments for specialised care such as mental health therapy. As mentioned by one refugee:

*“When I was having mental issues they referred me for mental therapy. But it wasn’t coming fast, it was taking time. They just toss you around, go to the GP, they will tell you, they will want you to call the number yourself. And when you call a number like talking therapy, they say they need to assess you, later they will tell you, oh, we can’t do this, you need to go somewhere else. It was difficult”*.

(Female refugee 16)

Although some healthcare providers were perceived as being racist because of the delays in obtaining appointments, they argued that the long waiting times for appointments applied not only to immigrants but to the general population as well. As such, one nurse practitioner recommended waiting patiently for appointments just like everyone else.

*“And it’s not because you’re an asylum seeker to wait sixteen weeks to see a physiotherapist. Joe Doe down the road is the same. And although it’s not a racist thing, you get this racist thing thrown in your face … but not a racist, we are in process, you just need to wait patiently”*.

(Nurse practitioner)

### 3.4. Affordability: Financial Barriers

Affordability emerged as a major barrier to healthcare access for homeless immigrants, and this challenge was shaped by the intersection of their immigration status, extreme poverty, homelessness, and limited entitlement to public funds, which collectively intensified their financial vulnerability within the healthcare system.

Several participants described experiencing unexpected charges for medications despite believing they were entitled to free prescriptions. These inconsistencies created confusion and distress, especially for individuals surviving on minimal government allowances. For many asylum seekers, information provided by GP practices was often unclear or contradicted the reality they encountered at pharmacies. Some were assured they qualified for free prescriptions, only to be charged later when collecting their medication. These unpredictable costs placed considerable strain on their already limited weekly stipends, forcing them to make difficult decisions about how to allocate their scarce financial resources. One female asylum seeker recounted a scenario:

*“Because we are asylum seekers, we are entitled to free medication and I’m not supposed to pay, but most times I end up paying for my medication … we called the GP and they said they will be giving me free medication. But the other time I went, I had to pay again. So, it’s not easy for me because I’m taking off of my weekly allowance”*.

(Female asylum seeker 27)

Financial barriers were also highlighted by stakeholders, who described how certain groups of immigrants, particularly those with no recourse to public funds (NRPF), faced additional costs despite being entitled to access NHS care. Although these individuals could register with a GP and receive primary healthcare, they were still required to pay prescription charges. For people already living with severe economic insecurity, this created significant obstacles to maintaining their health and adhering to treatment plans. As mentioned by one outreach service manager:

*“I think for people that have not got recourse, they have to pay for prescriptions. So, they’re entitled to the health care, but they have to pay for prescriptions”*.

(Outreach service manager)

Furthermore, stakeholders highlighted that NHS charging regulations for secondary care disproportionately affected homeless immigrants with NRPF, with healthcare costs accumulating rapidly and resulting in substantial debt. As a consequence, some GP practices restricted care to immediate pain management and declined to facilitate access to ongoing or rehabilitative therapies. This demonstrates how the intersection of immigration status and NHS charging policies constrained continuity of care and intensified vulnerability among homeless immigrants. One complex caseworker illustrated this through the following example:

*“A young man, with no access to public funds, and appeals rights exhausted, was hit by a car which gave him significant back pain. He was getting pain relief and other sorts of therapies. But he was starting to get charged for it and the charges built up into tens of thousands of pounds until his GP said they could not engage with him anymore. They refused him any sort of therapy intervention apart from immediate pain relief”*.

(Complex caseworker)

Immigrants with NRPF described a heavy reliance on friends, family, and charitable organisations to meet their basic needs. This dependence was experienced as profoundly disempowering and was associated with feelings of shame, vulnerability, and a loss of dignity and self-respect. Such experiences had a detrimental impact on mental health, with participants describing persistent emotional distress and depressive symptoms. As one failed asylum seeker explained:

*“When you are here and you have no recourse to public funds, it means you’re dependent on people, and friends, and it’s undignified. You have to ask people for certain basic things, in terms of how you dress, shoes, … you just feel vulnerable in that sense. There is no dignity, no respect, … So, it is depressing. It’s very depressing, you know”*.

(Male failed asylum seeker 13)

In addition, stakeholders noted that asylum seekers living on extremely limited daily allowances faced significant financial constraints, such that even modest travel expenses constituted a substantial barrier to accessing care. These costs often restricted their ability to attend appointments and engage in ongoing healthcare. This highlights how poverty and immigration status intersect to shape the ability to reach healthcare services, with transport costs operating as a practical yet consequential deterrent to access. As one refugee project officer explained:

*“Access to services could be challenged by travelling costs for asylum seekers because people who are seeking asylum live with less than 6 pounds a day. And that is to cover their needs … So that could prevent people from accessing services if they need to incur an additional cost because of the bus ride”*.

(Refugee project officer)

These financial barriers illustrate how overlapping identities, such as being an asylum seeker or NRPF, living in poverty and experiencing homelessness interact to deepen inequalities and restrict homeless immigrants’ ability to access consistent and equitable primary care services.

### 3.5. Appropriateness: Interactions with Healthcare Providers

Interactions with healthcare providers emerged as a key determinant of access to PCSs for homeless immigrants, with experiences shaped by the intersection of homelessness, immigration status, race, gender, and institutional attitudes, resulting in both supportive and exclusionary encounters. Participants expressed mixed views regarding their interactions with primary care providers, with experiences ranging from highly supportive to overtly exclusionary. These interactions played a critical role in shaping homeless immigrants’ access to, engagement with, and continuity of primary care services. Some participants described positive encounters with GPs and practice reception staff that facilitated access to primary care. Compassionate communication, active listening, and continuity of care were perceived as enabling trust and encouraging ongoing engagement with GP services despite housing instability. One refugee participant highlighted how a GP’s calm demeanour, extended consultation time, and proactive follow-up supported sustained access to care:

*“He was really good and calm. There are a lot of asylum seekers, and he was the favourite of the doctors. He was so calm if you had any problem, he always saw us, listened to what we’ve got to say, that GP was good… Sometimes he would see us for hours … If for example, he didn’t see me, he couldn’t get hold of me for a while, he had to call and check if I was okay, that’s how nice he was, you know, of all the doctors I saw”*.

(Female refugee 28)

Similarly, respectful and welcoming interactions with reception staff reduced anxiety around attending GP practices and supported navigation of appointment systems, thereby lowering administrative and psychological barriers to access. As one failed asylum seeker explained:

*“My experience with the GPs is very good … very good receptionists, very polite. They go the extra mile of trying to help you, to guide you … So that’s how they have been. And even the receptionist, when you go they want to have a chat with you, I mean, they are friendly anyway. I don’t how to put it but they are friendly”*.

(Male failed asylum seeker 13)

In contrast, other participants reported negative interactions within primary care settings that acted as barriers to access. Experiences of discrimination, stigma, and dismissive communication undermined trust in GP services and, in some cases, restricted appointment availability. Some participants felt that access to primary care was contingent on advocacy from charitable organisations, rather than recognised as a right, particularly for African and migrant patients. One refugee participant stated:

*“The discrimination is a lot, I am not gonna lie … I speak English, I don’t know how to speak my language, I was like you can understand me, why don’t you just give me an appointment, why? They would give their appointment if the charity was involved. I think there’s a stigma among people with a refugee or migrant background”*.

(Female refugee 28)

Additionally, disrespectful and judgmental encounters with GPs were described as exacerbating stress and discouraging further engagement with primary care services, particularly for homeless women experiencing multiple vulnerabilities. As one asylum seeker recounted:

*“She’s my doctor but she’s very rude, they respect no one, she always says, ‘You make me crazy. What do I do with you? Why not get married? Why not find a boyfriend?’ … She’s very rude”*.

(Female asylum seeker 4)

In addition, some participants described how being identified as homeless shaped their interactions with PCSs in ways that undermined access to care. Participants reported feeling stereotyped as unreliable or exaggerating symptoms, which led to their health concerns being dismissed or minimised by practice staff and clinicians. Such perceptions contributed to a lack of trust within clinical encounters and resulted in delayed assessment, inadequate treatment, and disengagement from GP services. One asylum seeker explained how assumptions linked to homelessness influenced how her pain was interpreted and managed in primary care:

*“Because they probably knew I am a homeless person … sometimes the management is rude. Most of the time when I go there to ask about my health problems, they think I’m doing some kind of drama … they think that I’m lying, so they don’t take it very seriously … They don’t trust us basically … For example, I have a very big back pain. Many times, I’ve asked my GP but he simply ignored me”*.

(Female asylum seeker 5)

These accounts illustrate how interpersonal interactions within primary care settings operated as both enablers and barriers, with intersecting social identities and structural positions shaping whether homeless immigrants were treated with trust, dignity, and responsiveness, or experienced stigma and dismissal that restricted access to care.

## 4. Discussion

This study provides an in-depth examination of how immigrants experiencing homelessness in England navigate access to PCSs, drawing on both lived experiences and stakeholder perspectives. Using Levesque et al.’s Patient-Centred Access to Healthcare Framework alongside an intersectionality lens, the findings reveal that access to PCSs is shaped less by individual health needs and more by the interaction of structural constraints and social positioning. Key barriers identified included the fear of detention and deportation, stigma surrounding mental health, digital exclusion, and financial barriers. These barriers were not experienced in isolation; rather, they accumulated at the intersections of homelessness, immigration status, poverty, racialisation, and gender, producing layered forms of exclusion from primary care. As a result, participants frequently perceived primary care not as a site of care and support, but as a space of risk, surveillance, and inaccessibility, highlighting how healthcare inequities are structurally produced and sustained within the UK’s healthcare and immigration systems.

A key insight from this study was the fear of detention and deportation that emerged as a significant structural barrier to accessing PCSs. Participants’ concerns about data sharing between healthcare providers and the Home Office led many homeless immigrants to perceive primary care settings as sites of surveillance rather than care. In this context of legal precarity, routine administrative procedures such as identity verification or address requirements became sources of heightened anxiety, actively discouraging healthcare seeking. Viewed through an intersectionality lens, this exclusion is not merely a consequence of irregular migration status in isolation. Rather, it emerges from interlocking precarities where the visibility of homelessness (lack of a stable address) converges with legal insecurity to amplify the individual’s exposure to the state. This creates a survival paradox, where homeless immigrants attempt to remain invisible to avoid detection, while the mandatory nature of GP registration forms hyper-visibilises their irregular status [32], triggering what participants described as a fear of detention and deportation. This tension is further exacerbated by the “hostile environment” policy landscape, specifically, the UK government’s 2021 designation of rough sleeping as potential grounds for deportation [33], which amplifies homeless immigrants’ fears of registering with GP practices. Consistent with wider evidence, irregular immigrants are disproportionately deterred from accessing NHS services and are three times more likely to fear arrest [34]. This asserts how the UK hostile environment policy hinders access to healthcare, undermining the principle that access to primary care should be independent of immigration or housing status [35].

Cultural norms and social expectations significantly influenced how participants interpreted and acted upon their health needs. Utilising an intersectionality lens reveals that mental health stigma did not operate in isolation; rather, it was intensified through its interaction with gendered expectations, particularly among men. For these individuals, the pressure to conform to traditional constructions of masculinity discouraged emotional expression, creating a barrier to accessing PCSs. This finding resonates with literature suggesting that, in an effort to preserve self-respect and maintain emotional stability, some immigrants adopt self-isolation as a coping mechanism to avoid the social shame associated with weakness or mental illness [36]. These intersectional stigmas often result in a paradox where health conditions are under-reported despite high levels of distress. Research by de la Calzada-Calugay and Hanley [37] indicates that stigma is a primary reason why some immigrant homeless populations report fewer instances of mental health issues, even when their actual health outcomes are poorer than their locally born counterparts’. This strategy was mirrored in the current study, where participants’ fear of social and institutional stigma directly hindered their willingness to seek help, ultimately leading to the concealment of serious mental health needs.

Organisational features of the healthcare system, particularly digitalisation, acted as a hinderance to accessing PCSs. From an intersectionality perspective, digital exclusion reflects a cumulative disadvantage in which poverty, housing instability, and social isolation combine to limit access to healthcare services. Evidence from the Good Things Foundation [38] shows that digital exclusion is closely linked to low income, meaning that digital access routes can create additional barriers for the most marginalised groups. This challenge is especially pronounced for individuals with NRPF, whose legal status restricts access to welfare support that might otherwise help offset digital and financial barriers [39]. In addition, although PCSs are free, financial insecurity continues to restrict access to care, as reported by participants in this study. Indirect costs such as paying for mobile data to conduct online consultations, covering transport to GP practices, and prescription costs often prevent access to PCS. This creates a cycle of exclusion, where poor health limits the ability to work, increasing financial hardship and housing insecurity, which in turn further reduces access to healthcare [40]. As de la Calzada-Calugay and Hanley [37] note, these pressures are particularly severe for migrants affected by healthcare charging policies or restricted access to public support. As a result, individuals are often forced to prioritise basic necessities such as food over medical care, meaning that healthcare access exists in principle but remains difficult to achieve in practice. These findings emphasise the limitations of policy approaches that focus narrowly on service availability without addressing the wider social and economic conditions that shape trust, safety, and capacity to engage with care. Improving access to primary care for this homeless immigrant population therefore requires not only clearer guidance and inclusive registration practices, but also structural reforms that dissociate healthcare from immigration enforcement, address digital and financial barriers, and recognise the lived realities of homelessness and irregular immigration status. Such changes are essential to uphold the principle of equitable access to healthcare and to prevent the continued reproduction of avoidable health inequalities.

### 4.1. Implications for Mental Health Promotion in a Digital Era

The findings of this study have important implications for public mental health promotion within an increasingly digital healthcare landscape. As the NHS accelerates its transition toward digital-first models [41], digital health innovations present a dual challenge: while they offer opportunities for mental health interventions, they also risk creating a new frontier of exclusion for populations experiencing deep digital poverty. Where individuals experiencing mental health issues were once physically separated from society in asylums, now they may experience a form of digital exclusion, limiting their ability to navigate healthcare systems, access timely care, or have their needs adequately recognised [42]. For immigrants experiencing homelessness, the barriers identified across the five dimensions of access suggest that this digital shift may systematically bypass those most in need.

The findings indicate that barriers to accessing PCSs may hinder the early identification and preventive management of mental health needs among homeless immigrants. Instead, the convergence of structural mistrust, legal precarity, and digital marginalisation shifts the trajectory of care-seeking away from primary prevention and toward emergency departments [43]. This produces a “crisis-led” model of care, in which individuals present only at advanced stages of psychological distress [44]. Such a reactive pattern not only undermines recovery trajectories but also places a disproportionate and avoidable burden on acute healthcare services. Improving how primary care supports mental health needs by providing culturally appropriate support, offering simple guidance to help people use digital services, and rebuilding trust is essential to ensuring that GPs can act as a reliable source of support [45]. Importantly, accessibility for some but not all undermines the core objective of digital mental health, which is to provide broadly equitable access, and risks reinforcing rather than reducing existing inequalities in care [46].

### 4.2. Strengths and Limitations

A key strength of this study is its qualitative research design, which draws on the lived experiences of immigrants experiencing homelessness alongside the professional insights of stakeholders involved in service provision. Including both groups enabled a more comprehensive understanding of how access to primary care is shaped not only by individual experiences but also by organisational practices and institutional norms. The study also benefits from the use of the Levesque Patient-Centred Access to Healthcare Framework in combination with an intersectionality lens, which allowed for a nuanced analysis of how multiple social identities, including immigration status, race, gender, and homelessness, intersect to shape access in ways that single-axis approaches often overlook. The study’s diverse sample, spanning various countries of origin, immigration statuses, and experiences of homelessness, strengthens the transferability of findings by demonstrating common patterns across different migrant groups. Despite these contributions, the study has limitations. All interviews were conducted in English, which may have excluded participants with limited language proficiency, potentially underrepresenting the experiences of those facing the greatest barriers. For both participant groups (i.e., homeless immigrants and stakeholders), female participants outnumbered male participants. This may have led to a slight overrepresentation of gendered experiences, although the impact of this imbalance is likely minimal. As the interviews with participants were conducted virtually, some non-verbal cues may have been missed, yet these can sometimes enrich the dialogue, particularly for participants for whom English is a second language. Lastly, although interviews lasting 45–60 min were considered sufficient and data saturation was achieved, the topic under investigation is emotionally sensitive, and some participants may have required additional time to fully express and process their experiences.

## 5. Conclusions

This study highlights how immigrants experiencing homelessness in the UK face multiple and intersecting barriers to accessing primary care services. By integrating the Levesque framework with an intersectionality lens, the findings show that systemic inequities, including digital exclusion, financial hardship, and culturally unresponsive care, significantly hinder access. These challenges contribute to delayed help-seeking and a shift toward crisis-driven use of emergency services, undermining both individual health outcomes and the efficiency of the healthcare system. From a public mental health promotion perspective, in a digital era where healthcare delivery is increasingly technology-driven, improving access to primary care for this population requires more than increasing service awareness or digital literacy. It calls for structural reforms that separate healthcare from immigration enforcement, reduce financial and administrative barriers, and ensure that digital transformation complements rather than replaces inclusive, low-threshold, and trust-based pathways to care. Without such changes, digital health innovations risk reinforcing existing inequalities and limiting equitable access to primary care for early mental health intervention and prevention. Ultimately, this study provides a strong evidence base to inform strategies for improving access to primary care in the digital era.

## Figures and Tables

**Table 1:** Characteristics of homeless immigrants.

Characteristics	*N* = 30	%
Gender		
Male	10	33.3
Female	20	66.7
Age group (years)		
18–29	7	23.3
30–39	9	30.0
40–49	8	26.7
50–59	5	16.7
≥60	1	3.3
Immigration status		
Asylum seeker	13	43.3
Refugee (including time-limited status)	9	30.0
Others (spouse, student, overstayer)	4	13.3
Failed asylum seeker	2	6.7
Indefinite leave to remain	2	6.7
Ethnicity		
African	21	70.0
Arab	4	13.3
Asian	3	10.0
Caribbean	2	6.7
Length of stay in the UK		
<5 years	3	10.0
5–9 years	11	36.7
10–14 years	6	20.0
≥15 years	9	30.0
Not reported	1	3.3

**Table 2:** Characteristics of stakeholders.

Characteristics	*N* = 30	%
Gender		
Female	21	70.0
Male	9	30.0
Voluntary sector roles (*n* = 16)		
Project workers	6	37.5
Managers	4	25.0
Mentoring coordinators	3	18.8
Youth ambassadors	2	12.5
Counsellor	1	6.3
Healthcare provider roles (*n* = 11)		
Nurse practitioners	4	36.4
General practitioners	3	27.3
Practice manager	1	9.1
Mental health specialist	1	9.1
Social prescriber	1	9.1
Specialist caseworker	1	9.1
Local council roles (*n* = 3)		
Social worker	1	33.3
Public health specialist	1	33.3
Rough sleeping manager	1	33.3

## Data Availability

The data presented in this study are available within the article.

## Supplementary Materials

The following supporting information can be downloaded at: https://www.mdpi.com/article/10.3390/ijerph23060726/s1, File S1: COREQ (Consolidated criteria for Reporting Qualitative research) Checklist.

## Abbreviations

The following abbreviations are used in this manuscript:

PCS	Primary Care Services
UK	United Kingdom
NHS	National Health Service
GP/GPs	General Practitioner(s)
A&E	Accident & Emergency
NRPF	No Recourse to Public Funds
