# Supplementary material for: Accessibility to Primary Care Services for Immigrants Experiencing Homelessness in England: A Qualitative Exploratory Study
Source: Int J Environ Res Public Health. 2026 May 29;23(6):726. doi: 10.3390/ijerph23060726 (PMC13299671; doi:10.3390/ijerph23060726)
Supplement: Supplementary file 1 [file ijerph-23-00726-s001.zip › ijerph-4298231-supplementary.pdf]

File S1. COREQ (Consolidated criteria for Reporting Qualitative research) Checklist .

Topic	Item No.	Guide Questions/Description	Reported on Page No.
**Domain 1: Research team and reflexivity**			
Personal characteristics			
Interviewer/facilitator	1	Which author/s conducted the interview or focus group?	Interviews were conducted by the first author (C.N.).
Credentials	2	What were the researcher's credentials? E.g. PhD, MD	The researcher held an MA in Public Health and was undertaking a PhD in Public Health at the time of the study.
Occupation	3	What was their occupation at the time of the study?	PhD student and researcher in Public Health.
Gender	4	Was the researcher male or female?	Female
Experience and training	5	What experience or training did the researcher have?	The researcher had training in qualitative research methods through PhD-level Public Health training and experience conducting qualitative interviews and thematic analysis.
Relationship with participants			
Relationship established	6	Was a relationship established prior to study commencement?	No prior relationship was established with participants before the study commenced. Some stakeholders were recruited through professional networks and organisations.
Participant knowledge of the interviewer	7	What did the participants know about the researcher? e.g. personal goals, reasons for doing the research	Participants were informed that the researcher was a PhD student conducting research on access to primary care services among immigrants experiencing homelessness in England. They were informed about the aims of the study and the researcher's interest in improving understanding of barriers to primary care access.
Interviewer characteristics	8	What characteristics were reported about the inter viewer/facilitator? e.g. Bias, assumptions, reasons and interests in the research topic	The interviewer was a female PhD researcher with training in qualitative public health research and an interest in health equity, migration, homelessness, and access to healthcare services. Reflexivity was maintained throughout the research process to minimise the influence of personal assumptions and biases on data collection and interpretation.
**Domain 2: Study design**			
Theoretical framework			
Methodological orientation and Theory	9	What methodological orientation was stated to underpin the study? e.g. grounded theory, discourse analysis, ethnography, phenomenology, content analysis	Pages 3-4
Participant selection			
Sampling	10	How were participants selected? e.g. purposive, convenience, consecutive, snowball	Page 4

Topic	Item No.	Guide Questions/Description	Reported on Page No.
Method of approach	11	How were participants approached? e.g. face-to-face, telephone, mail, email	Page 4
Sample size	12	How many participants were in the study?	Page 4
Non-participation	13	How many people refused to participate or dropped out? Reasons?	No participants withdrew after consenting to participate.
Setting			
Setting of data collection	14	Where was the data collected? e.g. home, clinic, workplace	
Presence of non participants	15	Was anyone else present besides the participants and researchers?	Page 4
Description of sample	16	What are the important characteristics of the sample? e.g. demographic data, date	Tables 1 and 2 on pages 6 and 7
Data collection			
Interview guide	17	Were questions, prompts, guides provided by the authors? Was it pilot tested?	Page 4
Repeat interviews	18	Were repeat inter views carried out? If yes, how many?	No repeat interviews were conducted.
Audio/visual recording	19	Did the research use audio or visual recording to collect the data?	Page 4
Field notes	20	Were field notes made during and/or after the inter view or focus group?	Yes. Field notes were made during and after interviews to capture and emerging reflections.
Duration	21	What was the duration of the inter views or focus group?	Page 4
Data saturation	22	Was data saturation discussed?	Page 5
Transcripts returned	23	Were transcripts returned to participants for comment and/or correction?	No. Transcripts were not returned to participants for comment or correction.
**Topic**	**Item No.**	**Guide Questions/Description**	**Reported on Page No.**
**Domain 3: analysis and findings**			
Data analysis			
Number of data coders	24	How many data coders coded the data?	Page 5
Description of the coding tree	25	Did authors provide a description of the coding tree?	Yes. Codes were reviewed for patterns and relationships, grouped into subthemes, and subsequently synthesised into broader overarching themes guided by the Levesque Framework and an intersectionality lens.
Derivation of themes	26	Were themes identified in advance or derived from the data?	Page 5
Software	27	What software, if applicable, was used to manage the data?	Page 5
Participant checking	28	Did participants provide feedback on the findings?	No. Participants did not provide feedback on the findings.

Topic	Item No.	Guide Questions/Description	Reported on Page No.
Reporting			
Quotations presented	29	Were participant quotations presented to illustrate the themes/findings? Was each quotation identified? e.g. participant number	Pages 7- 14
Data and findings consistent	30	Was there consistency between the data presented and the findings?	Yes. The findings were clearly grounded in the data, with consistent alignment between participant quotations and reported themes.
Clarity of major themes	31	Were major themes clearly presented in the findings?	Pages 7-14
Clarity of minor themes	32	Is there a description of diverse cases or discussion of minor themes?	Pages 7-14
